# Ultra-Wideband and Narrowband Switchable, Bi-Functional Metamaterial Absorber Based on Vanadium Dioxide

**DOI:** 10.3390/mi14071381

**Published:** 2023-07-06

**Authors:** Xiaoyan Wang, Yanfei Liu, Yilin Jia, Ningning Su, Qiannan Wu

**Affiliations:** 1 School of Information and Communication Engineering, North University of China, Taiyuan 030051, China; 2 Center for Microsystem Integration, North University of China, Taiyuan 030051, China; 15373166906@163.com (Y.L.); 13384612312@163.com (Y.J.); suningning826032@163.com (N.S.); 3 School of Instrument and Intelligent Future Technology, North University of China, Taiyuan 030051, China; 4 Academy for Advanced Interdisciplinary Research, North University of China, Taiyuan 030051, China; 5 School of Semiconductors and Physics, North University of China, Taiyuan 030051, China

**Keywords:** terahertz, metamaterial, perfect absorber, vanadium dioxide

## Abstract

A switchable ultra-wideband THz absorber based on vanadium dioxide was proposed, which consists of a lowermost gold layer, a PMI dielectric layer, and an insulating and surface vanadium dioxide layer. Based on the phase transition properties of vanadium dioxide, switching performance between ultra-broadband and narrowband can achieve a near-perfect absorption. The constructed model was simulated and analyzed using finite element analysis. Simulations show that the absorption frequency of vanadium dioxide above 90% is between 3.8 THz and 15.6 THz when the vanadium dioxide is in the metallic state. The broadband absorber has an absorption bandwidth of 11.8 THz, is insensitive to TE and TM polarization, and has universal incidence angle insensitivity. When vanadium dioxide is in the insulating state, the narrowband absorber has a Q value as high as 1111 at a frequency of 13.89 THz when the absorption is more excellent than 99%. The absorber proposed in this paper has favorable symmetry properties, excellent TE and TM wave insensitivity, overall incidence angle stability, and the advantages of its small size, ultra-widebands and narrowbands, and elevated Q values. The designed absorber has promising applications in multifunctional devices, electromagnetic cloaking, and optoelectronic switches.

## 1. Introduction

Metamaterials are a class of artificially fabricated microstructured materials with unique electromagnetic properties that cannot be achieved with natural materials. They are widely used in communication [1], imaging [2], stealth [3], sensing [4], and other fields. With the development of technology, the science and technology of terahertz have also led to the rapid growth of metamaterial devices. To facilitate the development of THz technologies, various metamaterial-based functional devices have been proposed, such as filters [5,6], polarization converters [7,8], modulators [9,10], antennas [11], and perfect absorbers [12,13]. Among these devices, metamaterial perfect absorbers have been a popular research topic due to their wide range of applications in solar energy, cloaking technology, etc. Most THz absorbers, however, have a single function. They cannot be dynamically tuned, etc., and actively tunable metamaterial absorbers are more suitable for complex electromagnetic applications in practical applications.

Metamaterials can be used in perfect absorbers based on metamaterials, which was first proposed by LANDY [14]. People have gradually introduced some active materials in the devices in order to be able to design tunable absorbers, such as vanadium dioxide [15,16], graphene [17,18,19,20,21], molybdenum disulphide [22,23,24], strontium titanate oxide [25,26], indium antimonide [27,28,29,30], etc. Among these phase-changing materials, vanadium dioxide can undergo a phase transition from an insulating state at room temperature to a stable high-loss metallic state at higher temperatures, and the optical and electrical properties can be significantly modified during the phase transition, which is reversible. As the conductivity of vanadium dioxide increases steadily when heated from room temperature to higher temperatures, vanadium dioxide can vary in the range of 20 S/m to 200,000 S/m [31,32,33]. Based on the fact that vanadium dioxide phase transitions require modest temperatures and can be accomplished at room temperature with few limitations in experimental testing and practical applications, vanadium dioxide is gradually being applied with these properties, and the optical properties of vanadium dioxide films can be altered by heating or cooling the films to temperatures close to the phase transition temperature. The phase-shift properties of vanadium dioxide are well suited to the tuning needs of metamaterial absorbers and are gradually being used more and more by researchers to prepare tunable metamaterial absorbers based on the phase-shift material vanadium dioxide [34,35]. In the last decade or so, metamaterial absorbers have been used in the preparation of virtual narrowband absorbers [36,37,38], broadband absorbers [39,40,41,42,43,44,45,46,47,48], and broadband and narrowband tunable absorbers.

For example, in 2021, Chunyu Zhang et al. proposed a dual-modulated broadband terahertz absorber based on vanadium dioxide and graphene, which could achieve more than 90% absorption from 1.04 THz to 5.51 THz with a broadband absorption of 4.07 THz [49]. In the same year, Zhipeng Zheng et al. proposed a terahertz perfect absorber based on an ultra-broadband flexible active switch with more than 90% absorption intensity at 8.5–11 THz, and it was switchable to a narrowband absorber by changing the vanadium dioxide conductivity [50]. In 2022, H Peng et al. proposed a broadband terahertz tunable multiple absorber based on phase-shift materials with a bandwidth of 5.5 THz in the range of 4.5–10 THz where the absorption intensity exceeds 90% [51]. In the same year, Pengyu Zhang et al. proposed an ultra-broadband tunable THz metamaterial absorber based on a double layer of vanadium dioxide in a square ring array with an absorption intensity in the 1.63 THz range and an absorption intensity exceeding 90% in the 12.39 THz range. The absorption bandwidth was 10.76 THz [52]. In 2023, Niujunhao et al. proposed a switchable bi-functional metamaterial based on vanadium dioxide for broadband absorption and broadband polarization in the terahertz band, with absorption intensity exceeding 90% in the range of 3.3–5.62 THz [53]. Peng Gao et al. also proposed a broadband terahertz polarization converter based on the phase transition properties of vanadium dioxide in the same year, with a bandwidth of 2.87 THz in the range of 2.71–5.58 THz where the absorption exceeds 90% [54]. In 2021, Zhangbo Li et al. proposed an “Ultra-narrow-band metamaterial perfect absorber based on surface lattice resonance in a WS_2_ nanodisk. Array”, which has a sensitivity of 1067 nm/RIU when used as a narrowband absorber [55]. In 2022, Xianglong Wu et al. proposed a “High performance dual-control tunable absorber with switching function and high sensitivity based on. Dirac semi-metallic film and vanadium oxide”, which has a sensitivity of 462 GHz/RIU as a narrowband absorber [56]. Although there have been numerous studies of broadband absorbers for several years, terahertz absorbers that combine broadband absorption and narrowband absorption with a wider band and a simple structure have occasionally been reported. Moreover, currently reported broadband absorbers and narrowband switchable absorbers suffer from issues such as insufficient absorption bandwidth to meet practical applications and low Q values when switching to narrowband absorbers.

This paper proposes a dual-function THz metamaterial absorber based on vanadium dioxide for ultra-broadband and ultra-narrowband switching. The absorber size is considerably smaller than all current absorbers, making it more suitable for practical applications. When vanadium dioxide is in the metallic state, the absorber behaves as a broadband absorber with more than 90% absorption in the 3.8–15.8 THz range. When vanadium dioxide is in the insulating state, the absorber can be switched to a narrowband absorber with elevated Q values. The absorption at 13.89 THz is 99.99% with a Q value of 1111. Due to the extreme symmetry of the designed absorber, the absorber also has the characteristics of polarization insensitivity and insensitivity within a wide incidence angle of 50°, which considerably reduces the limitations of the absorber in a practical application. The ultra-wide and ultra-narrow bifunctional absorbers designed in this paper can provide current research ideas for versatile and tunable devices in the terahertz and its infrared bands, with promising applications in terahertz imaging, detection, and sensing.

## 2. Design and Simulation

**Figure 1:**
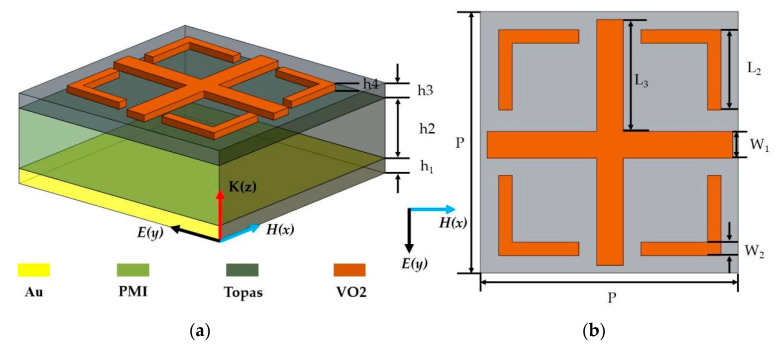
(**a**) 3D schematic of a terahertz metamaterial absorber; (**b**) top view of the *x–y* plane.

The structure diagram of our proposed vanadium-dioxide-based switchable absorber unit cell is shown in Figure 1, which consists of four layers: the lowermost structure is 0.2 µm of thick gold, which acts as a reflecting mirror to guarantee the complete reflection for the impinging terahertz wave, thereby suppressing the transmission; the second layer is a PMI (polymethacryl imide) layer with a relative permittivity of 1.1 [57]; the third layer is an insulating layer (Topas (cyclic olefin copolymer)) with a relative permittivity of 1.96, which is assumed to be lossless [58]; and the uppermost layer is a vanadium dioxide layer with a thickness of 200 nm. Figure 1b shows a top view of the unit cell structure with period p. The uppermost layer structure consists of a cross-like structure and four L-shaped dart-like structures. The optimal geometrical parameters were determined by analyzing the effect of the geometrical parameters on the absorption broadening, as shown in Table 1.

**Figure 2:**
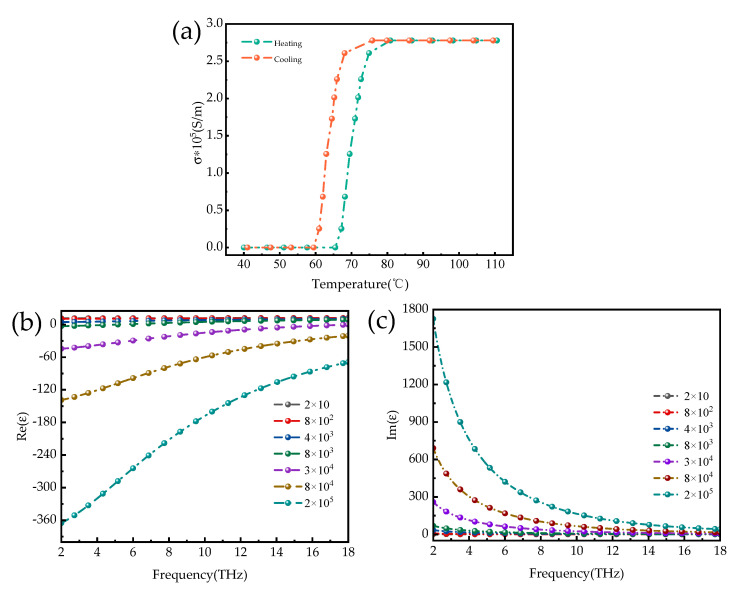
(**a**) The curve of VO_2_ conductivity as a function of ambient temperature; (**b**,**c**) real and imaginary parts of the relative permittivity of VO_2_ at different electrical conductivities.

**Figure 3:**
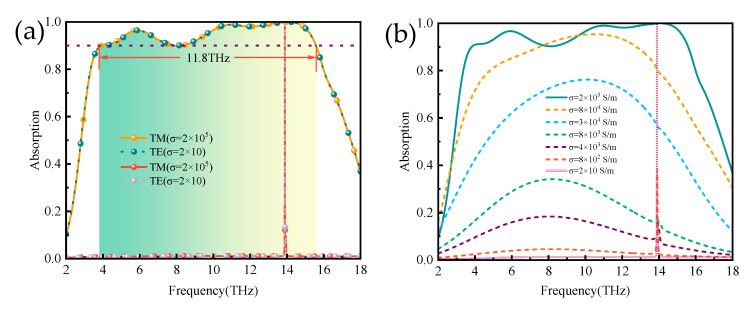
(**a**) Broadband absorption curve of VO_2_ in metallic phase (green line) and narrowband absorption curve of VO_2_ in insulating phase (pink line); (**b**) plots of absorptivity for absorbers with different conductivities.

In this paper, we numerically simulate the absorption properties of a designed switchable metamaterial absorber using the CST software. When a THz wave is vertically incident on the surface of a metamaterial absorber, the electric field of the incident electromagnetic wave is polarized in the x-direction, and the magnetic field is polarized in the y-direction. The structure of the absorbing metamaterial receiver cell extends indefinitely in the *x–y* plane. The Drude model describes the dielectric constant of *VO*_2_ in the THz range, where the dielectric constant of *VO_2_* can be expressed as [59]:

(1) $$\varepsilon {\left(\omega \right)}_{VO_{2}}=\varepsilon_{\infty }-\frac{\omega_{p^{2}}^{2}}{\omega {\left(\omega +i\gamma_{2}\right)}^{}},$$

where $\varepsilon_{\infty }$ = 12 is the permittivity at infinite frequency for vanadium dioxide, $\gamma_{2}$ = 5.75 × 10^13^ rad/s is the collision frequency, and $\omega_{p^{2}}$ is the plasmon frequency which depends on the conductivity *σ*. The relation between them may be stated as follows:

(2) $$\omega_{p^{2}}^{2}=\frac{\sigma }{\sigma_{0}}\omega_{p^{0}}^{2},$$

with *σ*_0_ = 3 × 10^5^ S/m and $\omega_{p^{2}}^{2}$ = 1.4 × 10^5^ S/m, the conductivity σ can vary with the phase transition of VO_2_. When the temperature changes, VO_2_ can switch back and forth between the insulating and metallic states [60], and the change in conductivity is reversible with the change in temperature. The variation in the conductivity of vanadium dioxide with ambient temperature is shown in Figure 2. Vanadium dioxide is insulated at room temperature and has an electrical conductivity of 20 S/m. When the temperature gradually increases, vanadium dioxide reaches the phase transition state, and the rice dioxide is in a metallic state with an electrical conductivity of 2 × 10^5^ S/m; moreover, the phase transition state of vanadium dioxide is reversible. When the ambient temperature is lowered, the metallic state can be transformed into an insulating state. The variation in the conductivity of vanadium dioxide at different temperatures is mainly due to the effect of temperature on the permittivity. Figure 3 shows the real and imaginary parts of the permittivity at different conductivities of vanadium dioxide. As shown in Figure 3, the imaginary part of the permittivity of vanadium dioxide is considerably larger than the real part, and the rate of change in conductivity at the transition is also larger than the real part. In practice, the following two methods can be used to make the vanadium dioxide phase transition. The first method is to heat the metal at the bottom of the absorber and convert the vanadium dioxide from an insulating to a metallic state by heat transfer, at which point the conductivity gradually increases. When the heating source is removed, the temperature gradually decreases, and the conductivity of vanadium dioxide gradually decreases; the metallic titanium transitions to an insulating state. The second method is to add a metallic patch to the vanadium dioxide layer and transfer heat to the vanadium dioxide by applying a voltage at the two ends of the metal, thus changing it from an insulating to a metallic state. When the voltage at both ends is removed, the temperature gradually decreases, and the vanadium dioxide transitions from a metallic to an insulating state. In addition, there are chemical mixing methods, etc., which can enable the phase transition of vanadium dioxide from the insulating to the metallic regime. With the above method, we can change the state of vanadium dioxide in practical applications so that the absorber designed in this paper can switch between ultra-widebands and ultra-narrowbands, enabling multi-functional device applications.

In this paper, we use the finite element theory to obtain ultra-narrowband and ultra-wideband absorption spectra of the proposed THz metamaterial absorber in the insulating and metallic states of vanadium dioxide. In the simulations, the absorption rate can be expressed as follows.

(3) A=1−R−T,

In Equation (3) where *A*, *T*, and *R* above are the absorptance, transmittance, and reflectance of the absorber, respectively. *R* = |S_11_|^2^ and *T* = |S_21_|^2^. |S_11_| and |S_21_| represent the reflection and transmission coefficients of the metamaterial absorber, respectively. In this paper, the structure has an underlying metal thickness of 0.2 μm, which is larger than the skin depth of THz waves at the target frequency, and the transmittance *T*(ω) is close to 0, which is simplified as *A* = 1 − *R* [61].

## 3. Results and Discussion

Through simulation, we calculated the absorption spectra of the absorber at different polarization modes (TE, TM) and different conductivities, respectively, as shown in Figure 3. From Figure 3a, when the VO_2_ is in the metal phase with a conductivity of 2 × 10^5^ S/m, the absorption bandwidth of more than 90% absorption is observed to be 11.8 THz in the frequency range from 3.8 THz to 15.6 THz, with a central frequency of around 9 THz, and the absorber has the advantage of being polarization insensitive. When VO_2_ undergoes a phase transition from a metallic to an insulating state under the influence of temperature, the absorber switches from a broadband absorber to a narrowband absorber with elevated Q values. The absorber achieves an absorption of >99.99% at the electromagnetic frequency f = 13.89 THz with a quality factor of 1111.

The absorption spectra of different conductivities are shown in Figure 3b. The figures show the switching function of the absorber in the broadbands and narrowbands as the conductivity of vanadium dioxide varies from 2 × 10 S/m to 2 × 10^5^ S/m. Upon heating, the vanadium dioxide transitions from an insulating to a metallic state while the absorber forms a conventional metal–dielectric–metal structure. Broadband absorbers enable switching between total reflection and perfect absorption. The absorber designed in this paper implements a switchable function between broadband and narrowband, which can also be referred to as the on–off function of a THz absorber.

**Figure 4:**
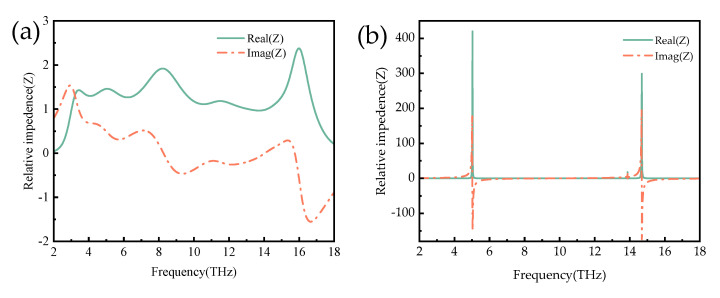
(**a**) Normalized impedance of the structure at σ = 2 × 10^5^ S/m. (**b**) Normalized impedance of the structure at σ = 2 × 10 S/m.

To further explain the absorption principle of the designed absorber, the impedance matching theory is used. The relative impedance formulae are shown in Equations (4) and (5) [62,63].

(4) $$A=1-R=1-{\left|\frac{Z-Z_{0}}{Z+Z_{0}}\right|}^{2}=1-{\left|\frac{Z_{r}-1}{Z_{r}+1}\right|}^{2},$$

(5) $$Z_{r}=\pm \sqrt{\frac{{\left(1+S_{11}\right)}^{2}-S_{21}^{2}}{{\left(1-S_{11}\right)}^{2}-S_{21}^{2}}},$$

where S_11_, S_21_, Z, and Z_0_ are the S-parameters, effective impedance and free space impedance of the proposed absorber, respectively, and Zr = Z/Z_0_ represents the relative impedance. Formulas (4) and (5) can be obtained to make the metamaterial absorber x absorption rate reach the maximum. At this time, in free space impedance matching, Z and Z_0_ represent the equivalent impedance and free space impedance of the absorber, while Z_r_ represents the relative impedance (Z_r_ = Z/Z_0_ = 1); Figure 4 shows the real and imaginary parts of the relative impedance of the metallic and insulating absorber under TE polarization. In Figure 4a, when VO_2_ is in the metallic state, the real part is close to 1, and the imaginary part is close to 0 in the frequency range of 3.8~15.6 THz. In this frequency range, the absorber’s impedance and the free’s impedance are already adequately matched. When the electromagnetic wave is incident to the absorber, the reflected wave is approximately 0, and most of the energy is lost in the insulating layer, thus achieving perfect absorption.

**Figure 5:**
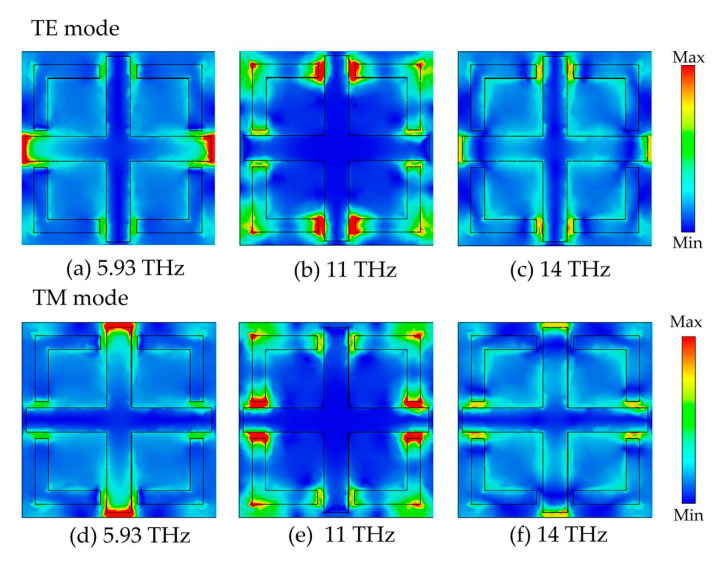
The electric field intensity distribution of the designed metamaterial wideband absorber at a frequency of 5.93 THz, 11 THz, and 14 THz; (**a**–**c**) for the TE mode and (**d**–**f**) for the TM mode.

In order to further explain the specific mechanism of the proposed absorber in wideband absorption, Figure 5 shows the wideband absorption field distribution of metal vanadium dioxide absorbent at different resonant frequencies in TE and TM polarization states. Figure 5a–c show the electric field distribution at frequencies of 5.93, 11, and 14 THz in TE mode. It is observed that the absorber exhibits a strong electric field at 3.5 THz at the left and right ends of the crossover, resulting in strong THz trapping and absorption. This means that the first absorption peak is caused by the coupling effect between two neighboring cells. When the frequency is 11 THz, the electric field is distributed in the horizontal gap between the upper and lower ends of the cross and the L-shaped dart. It is implied that the second absorption peak is caused by the interaction between the cross and the L-shaped dart. At 14 THz, the electric field strength of the vanadium dioxide resonant structure is particularly weak compared to the first two structures, indicating that the interaction between the vanadium dioxide surface structure and the incident terahertz wave is weak at this time. Most of the terahertz wave is lost in the dielectric layer at this time, thus producing absorption. Figure 5d–f show the electric field distribution of the absorber in TM mode at 1.93, 11, and 14 THz, which is similar to Figure 5a–c. Due to the perfect symmetry of the absorber designed in this paper, the absorber exhibits absolute polarization insensitivity. Therefore, the electric field distribution of absorber z is the same in both polarization states but with a 90° rotation.

**Figure 6:**
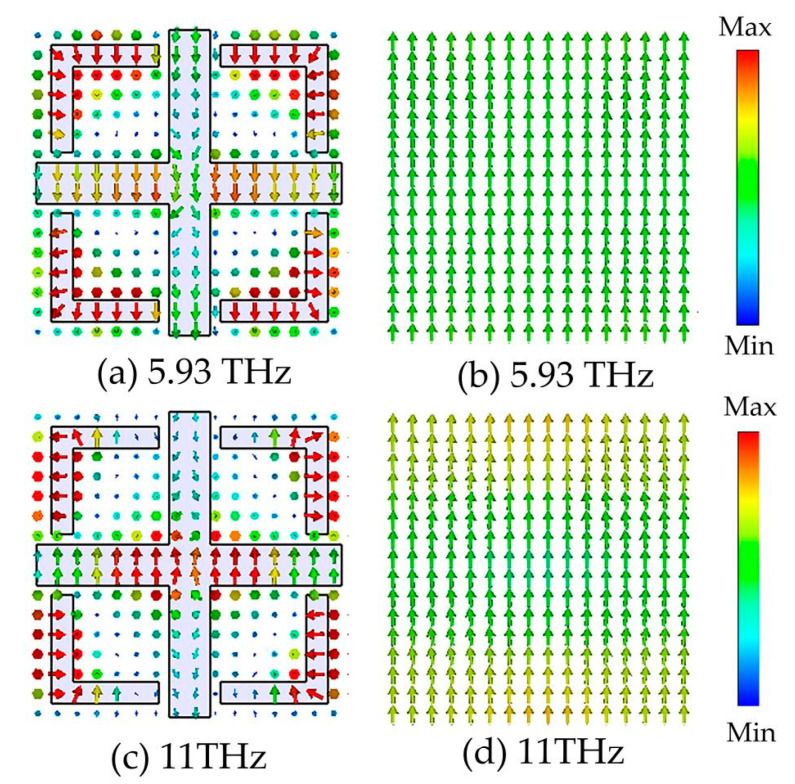
Distributions of the surface current on the top VO_2_ resonant ring and bottom metal layer at the frequencies of 5.93 THz and 11 THz under normal incidence of TE incident wave.

Figure 6 shows the current distribution on the surface of the top VO_2_ resonant layer and the bottom metallic layer at two resonant frequencies of 5.93 and 11 THz when VO_2_ is in the metallic state. At 3.5 THz, the direction of the surface current on the top VO_2_ resonance structure is inversely parallel to the direction on the bottom metal layer. The middle part of the dielectric layer is treated as a magnetic dipole, forming a strong magnetic resonance. At a frequency of 11 THz, a portion of the surface current direction on the top VO_2_ resonant structure is parallel to that on the bottom metal layer, when an electrical resonance is also induced. Thus, under the excitation of magnetic and electrical resonances, the incident electromagnetic waves are extremely suppressed in the resonator, leading to broadband absorption properties.

**Figure 7:**
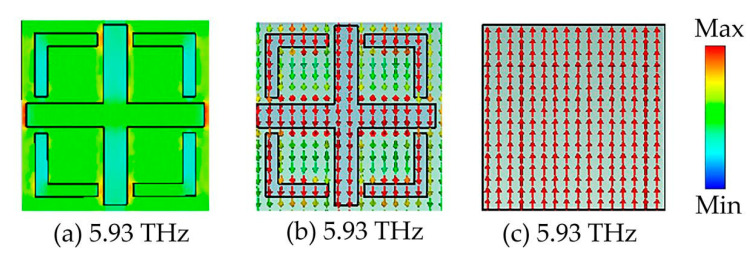
Distribution of electric fields and currents at 13.88 THz in narrowband absorption; (**a**) electric field distribution diagram; (**b**) surface current distribution diagram; (**c**) bottom surface current distribution diagram.

Figure 7 shows the electric field and current distribution of the absorber as a function of the narrowband absorber at 13.88 THz when vanadium dioxide is in the insulating state. As shown in Figure 7a, at a frequency of 13.88 THz, the electric field is mainly concentrated in the middle of the horizontal gap between the left and right ends of the cross-shaped structure and between the upper and lower ends of the cross-shaped and L-shaped darts. The narrowband absorption in this case is due to the coupling effect between the components. When an external electromagnetic wave interacts with the vanadium dioxide dielectric layer, the vanadium dioxide layer acts as a resonator, causing charges to accumulate in the surface structure and forming electric dipole resonances. This induced electric dipole resonance couples to the underlying metal plate, resulting in the formation of a magnetic dipole resonance in the absorber, which leads to a solid magnetic resonance producing a resonant absorption peak at 13.88 THz. Figure 7b,c show the surface and bottom metal current distributions of vanadium dioxide in the narrowband absorption state and the insulating state. The current direction at the top of the vanadium dioxide is parallel to the current direction at the bottom of the metal, forming a loop. This current distribution further validates the theoretical analysis section. In addition, the vanadium dioxide layer acts as a dielectric layer, where the thickness of the dielectric layer increases, providing a useful space for electromagnetic wave propagation and results in a narrowband peak of near-perfect absorption.

**Figure 8:**
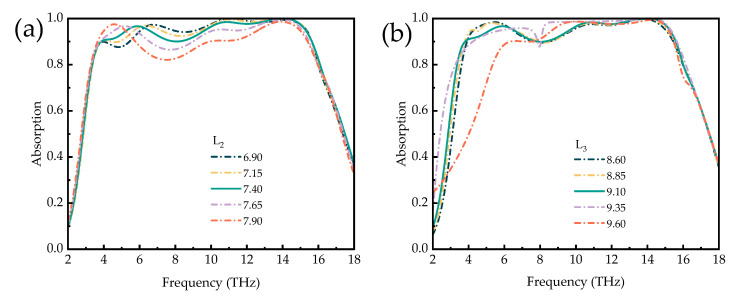
Influence of the (**a**) length L_2_ of the cross-like structure and (**b**) length L_3_ of the L-shaped, dart-like structure based on VO_2_ on the absorption spectrum.

**Figure 9:**
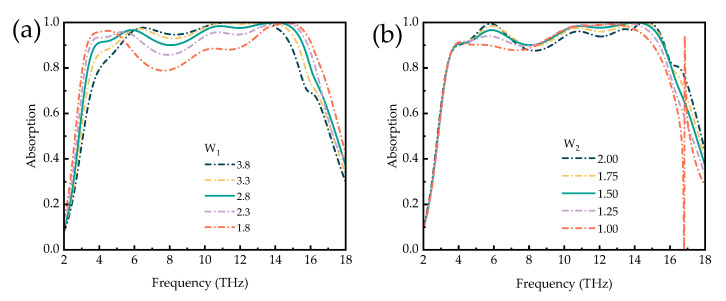
Influence of the (**a**) length W_1_ of the cross-like structure and (**b**) length W_2_ of the L-shaped, dart-like structure based on VO_2_ on the absorption spectrum.

To investigate the effect of the geometric structure parameter on the results, it is necessary to verify the effect of the geometric structure parameter on the absorber. One parameter is analyzed for simplicity, while the additional parameters are fixed. Figure 8 shows the effect of the length of the cross-shaped structure and the length of the L-shaped dart on the absorption spectra, respectively. In Figure 8a, as the L-shaped dart length b keeps increasing, the absorption spectrum shifts slightly at low frequencies, but the absorption intensity gradually decreases. Finally, the x absorption spectrum with a maximum absorption more significant than 90% is obtained at L_2_ = 7.4 μm. In Figure 8b, with the increasing length of the cross-type, the absorption peak at low frequencies gradually shifts blue, and the absorption intensity gradually decreases with the increasing length, which is caused by the coupling effect between adjacent unit structures. The effects of the cross-shaped structure k-width and L-shaped dart width on the absorption spectra are shown in Figure 9a,b, respectively. In Figure 9a, with the increasing length of W_1_, the absorption peaks with >90% absorption at low and high frequencies are red-shifted and blue-shifted, respectively, but the absorption intensity gradually increases, and finally, the absorption broadband is at its maximum at W_1_ = 2.8 μm. In Figure 9b, with the gradual increase in the L-shaped dart width, the position of the absorption peak at elevated frequency gradually shifts blue, and the absorption intensity gradually decreases. In summary, the best effect of the absorption bandwidth can be obtained by adjusting the geometric parameters of the structure. It will have important implications for practical fabrication.

**Figure 10:**
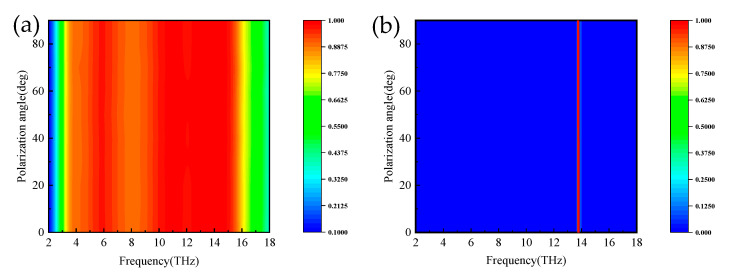
The absorption spectra of broadband absorbers in TM and TE modes; (**a**,**b**) different polarization angles.

**Figure 11:**
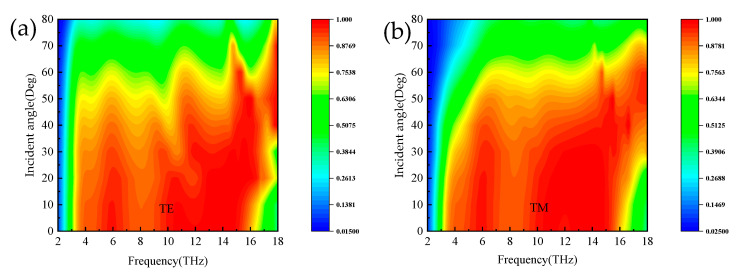
The absorption spectra of broadband absorbers in TE and TM modes; (**a**,**b**) different incident angles.

**Figure 12:**
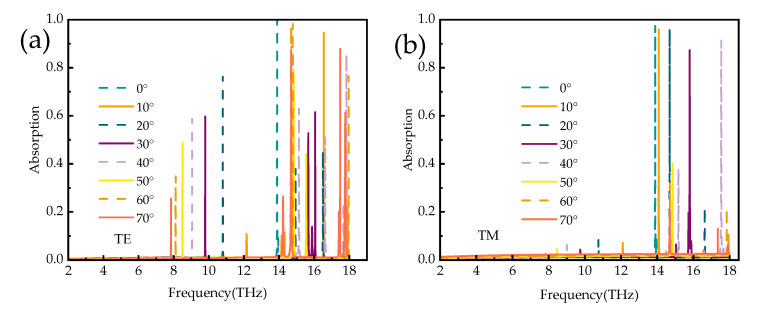
The absorption spectra of narrowband absorbers in TE and TM modes; (**a**,**b**) different incident angles.

In practice, a major factor in evaluating absorber properties is the absorption properties at different polarization angles and incidence angles. Figure 10a,b show the absorption spectra of the proposed absorber for normal incident waves at broadband and narrowband with polarization angles ranging from 0° to 90°. As seen from the plots, the absorption spectrum remains constant; thus, this absorber has the advantage of polarization insensitivity. Figure 11a,b show the absorption spectra of the broadband absorber under TE and TM polarized waves, respectively. When the incidence angle is less than 25°, the absorber has superior absorption characteristics in the range of 3.8–15.6 THz. The blue shift occurs when the incidence angle is 25° to 52°, with superior absorption characteristics in the field of 3.8–18 THz. In the TM mode in Figure 12b, when the incidence angle is greater than 40°, the absorption rate will blue-shifted; however, when the incidence angle is less than 55°, the absorber shows superior absorption characteristics. The absorption rate of the absorber in the TM and TE modes will be blue-shifted with the increasing incidence angle. The main reason is that the tangential component of the electric field decreases as the incidence angle increases. Remarkably, the bandwidth of the incidence angle widens with increasing incidence angle. This is because the TE polarization produces different absorption peaks at extreme frequencies, resulting in the broadening of the absorption bandwidth. For TM polarization, the absorption peak is significantly blue-shifted at high frequencies, resulting in a wider absorption bandwidth. Figure 12 shows the absorption spectra of the narrowband absorber in TE and TM modes at different incidence angles when vanadium dioxide is in the insulating state. In Figure 12a, with the increase in the incidence angle, the absorption spectrum gradually blue-shifts, the absorption rate is lower at the low frequency, and the absorption rate is higher in the range of 60°. In Figure 12b, the absorption spectrum also blue-shifts with increasing incidence angle in TM mode and maintains a high absorption peak in the range of 50°. As a narrowband absorber, it has a high incidence angle to maintain a high absorption rate in both modes, which increases the application value of the device.

**Figure 13:**
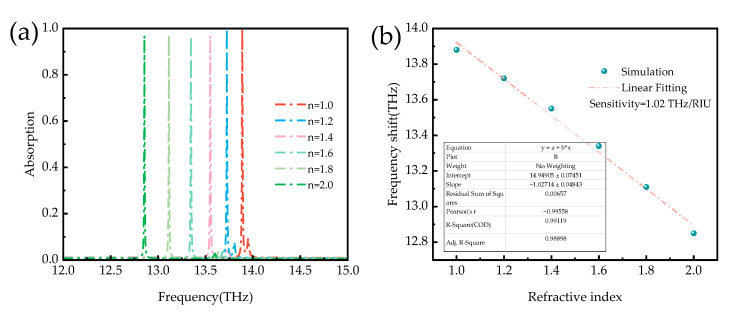
(**a**) Absorption spectra with different refractive index values of the surrounding dielectric environments; (**b**) the frequency shift against different refractive indices.

Since the sensor designed in this paper is a broadband and narrowband tunable absorber, we additionally evaluated the performance of the narrowband sensor absorber to consider practical applications of the sensor. Figure 13 shows the absorption spectrum of the narrowband absorber as a function of the refractive index of the surrounding environment. Figure 13a shows that the position of the narrowband absorption peak is red-shifted with the increasing refractive index. Sensitivity (*S*) is commonly used to evaluate the sensing performance of narrowband absorbers. In general, the sensor sensitivity is defined as shown in Equation (6). In addition, the FOM value reflects the influence factor bandwidth, which is also one of the critical metrics to reflect the sensor performance and is calculated as shown in Equation (7).

(6) S=Δf/Δn,

(7) FOM=S/FWHM,

In Equation (6), Δ*f* is the peak absorption shift due to the refractive index (Δ*n*) change. From Figure 13, it can be seen that when the refractive index gradually increases, the absorption peak of the sensor gradually red-shifts. After fitting the sensitivity of the sensor calculated as 1.02 THz/RIU, and with the increase in the refractive index, the absorption of the sensor is above 95% and also has the performance of a high Q value. In Equation (7), the FWHM is the full width at a half-height maximum of the absorption peak, and the higher the FOM value, the better the performance of the representative sensor and the higher the sensing accuracy, which can be calculated from the FOM value of the designed narrowband absorber of 102. Therefore, the narrowband absorber proposed in this paper can be used for detection by detecting changes in the refractive index of the analyte and has shown superior performance in subsequent bio-detection and sensing.

**Figure 14:**
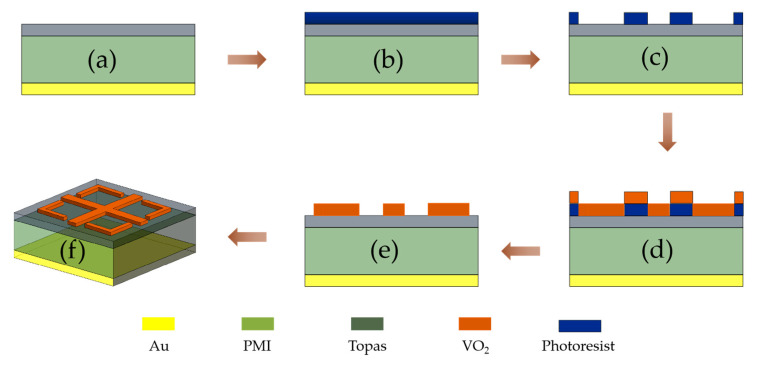
Absorber process flow diagram; (**a**) preparation of the bottom gold and PMI dielectric layers; (**b**) photoresist coating; (**c**) exposure, development; (**d**) sputtered precipitated vanadium dioxide layer; (**e**) release photoresist; (**f**) absorber samples.

This section presents the specific process and fabrication steps of the metamaterial absorber, which is shown in Figure 14. Due to the limited size of the terahertz band material units, most of them use micro-nano processing. These include the laser lithography process, inkjet printing process, MEMS process, etc. Due to the extreme accuracy of THz metamaterials, the resonant structure of the top unit cell of the metamaterial is fabricated in this work with laser etching. In Figure 14a, a layer of the metal film is sputter-grown on the back of the PMI layer after 0.2 μm using magnetron sputtering, and then a Topas insulating layer is superimposed on the PMI in the same way. Figure 14b shows a layer of photoresist uniformly spin-coated on the washed and air-dried prototype and dried at a suitable temperature. Figure 14c the photoresist is exposed to UV light under the premise that the sample is aligned with the mask plate, the exposed sample is carefully put into the developing solution and dried, and the pattern of the mask plate will appear on the developed sample. Figure 14d shows the sputtering of vanadium dioxide onto the developed sample in c using magnetron sputtering. Figure 14e shows the sample immersed in acetone solution to remove the excess photoresist, resulting in the designed vanadium dioxide resonance pattern. After the above process, a sample of the metamaterial absorber is obtained as shown in Figure 14f.

In Table 2, we compare the performance of the designed metamaterial absorber with alternative absorbers. The metamaterial absorber designed in this paper not only enables the switchable functionality of both broadband and narrowband absorbers, but it also has excellent absorption performance when the absorber is in the broadband regime, with a wider range of absorption than previously reported. When the absorber is in the narrowband, not only does it have the advantage of high Q values, but it also detects the ambient refractive index. Moreover, the metamaterial absorber designed in this paper has the advantages of modest size, simple structure, and easy handling.

## 4. Conclusions

In summary, we have designed an ultra-broadband and ultra-narrowband switchable, bi-functional THz absorber based on vanadium dioxide. The phase-shifted nature of vanadium dioxide, which modifies the conductivity with temperature, enables the switching of metamaterial device functions. When vanadium dioxide is in the metallic state, the absorber can be used as an ultra-wideband absorber, providing superior broadband absorption performance with an absorption rate of more than 90% in the 3.8–15.6 THz range and an absorption bandwidth of 11.8 THz. When vanadium dioxide is insulating, the designed absorber switches from an ultra-broadband absorber to an ultra-narrowband absorber. The designed sensor is a refractive index sensor to simulate different refractive indices in the ambient medium. The sensitivity of the proposed narrowband absorber was found to be 1.02 THz/RIU. Therefore, the proposed narrowband absorber can probe the refractive index by observation and sensing, and different devices exhibit excellent performance. The metamaterial absorber designed in this paper also has the properties of polarization insensitivity, wide incidence angle, small size, elevated Q value, and easy handling. The design has potential applications in terahertz imaging, electromagnetic stealth, and small optoelectronic switches.

## Figures and Tables

**Table 1:** Parameters of the designed absorber.

Parameter	P	h_1_	h_2_	h_3_	h_4_	W_1_	W_2_	L_2_	L_3_
Value/μm	22	0.2	6.55	0.15	0.2	2.8	1.5	7.4	9.1

**Table 2:** The performance of the proposed absorber is compared with that of recent years.

Reported Year and Reference	FB > 90 (THz)	BW > 90 (THz)	Materials	Functions (Absorption Band)	Layers
2021 [49]	1.04–5.51	4.47	Graphene and VO_2_	narrowband	5
2021 [50]	8.5–11	3.5	VO_2_	narrowband and broadband	3
2022 [51]	4.5–10	5.5	VO_2_	narrowband	4
2022 [52]	1.63–13.39	10.06	VO_2_	narrowband	6
2023 [53]	3.3–5.62	3.32	VO_2_	narrowband	6
2023 [54]	2.63–5.27	2.64	VO_2_	narrowband	3
This work	3.8–15.6	11.8	VO_2_	narrowband and broadband	4

## Data Availability

The data that support the findings of this study are available from the corresponding author upon reasonable request.
